# Green Synthesis of Nanoparticles in Mitigating Postharvest Losses of Fruits and Vegetables

**DOI:** 10.1002/fsn3.70017

**Published:** 2025-02-10

**Authors:** Ayesha Shakeel, Madiha Rohi, Rizwana Batool, Saima Tehseen, Mahwash Aziz, Kaynat Malik, Mahreen Abdul Sattar, Awais Raza, Agoura Diantom

**Affiliations:** ^1^ Department of Food Science and Technology Government College Women University Faisalabad Pakistan; ^2^ Faculty of Allied Health Sciences, University Institute of Food Science and Technology The University of Lahore Lahore Pakistan; ^3^ Department of Food Science and Technology Universite de Lomé Lome Togo

**Keywords:** fruits and vegetables, green synthesis, silver nanoparticles, statistical analysis, titratable acidity

## Abstract

The green synthesis of silver nanoparticles (AgNPs) from biological waste is an emerging technology that has excellent antibacterial properties. The present study has been designed to prepare silver nanoparticles by adopting green synthesis, which is based on the drying of fruits and vegetable peel to form silver nanoparticles. Two types of fruits (apples and tomatoes) and three types of vegetables (carrots, capsicum, and cucumber) were divided into three groups: one group was kept without any treatment, the second group was subjected to nanoparticles without silver nitrate, and the third group was subjected to silver nanoparticles. All the groups were stored for 15 days at room temperature and assessed for the physiochemical analysis of fruits and vegetables at 0 and 15th day and weight loss at 0, 5, 10, and 15th day of storage. Specifically, the titratable acidity of apples increased from 1.45 to 1.47 g/L, whereas nanoparticles and silver nanoparticles‐treated apples ranged from 1.40 to 1.43 g/L. For tomatoes, the titratable acidity decreased from 0.54 to 0.44 g/L in controls, compared to 0.39–0.44 g/L in treated samples. Carrots in the control group decreased from 0.38 to 0.32 g/L, whereas treated samples maintained 0.29–0.34 g/L. Capsicum's acidity fell from 0.37 to 0.27 g/L in controls, compared to 0.28–0.32 g/L in treated capsicum. Cucumber's acidity decreased from 0.23 to 0.17 g/L in controls, whereas treated cucumbers showed 0.40–0.46 g/L. Overall, the nanoparticle treatments were effective in preserving the produce's titratable acidity, indicating enhanced freshness and extended shelf life. It was examined that treatments treated with nanoparticles and silver nanoparticles have a great impact on the shelf life of fruits and vegetables. There is a great possibility of using nanoparticles and silver nanoparticles in combination with peel extract of fruits and vegetables to improve the shelf life of vegetables and fruits.

## Graphical abstract

The utilization of green chemistry principles in the synthesis of nanoparticles from fruits and vegetable peel waste has emerged as a promising approach to environmental sustainability. In this study, silver nanoparticles were prepared through a green synthesis approach using dried fruits and vegetable peels. The fruits and vegetables were divided into three groups: untreated, treated with nanoparticles without silver nitrate, and treated with silver nanoparticles. The results indicate that treatments involving nanoparticles and silver nanoparticles significantly impacted the shelf life of fruits and vegetables.

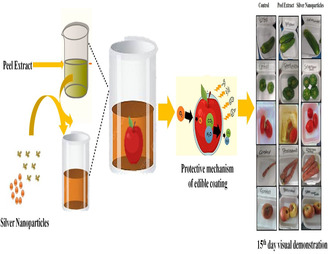

## 1 Introduction

Over the past few years, there has been a 30% growth in the global output of fresh fruits and vegetables (Hess and Sutcliffe 2018). There is gradual increase in production and hence the increment in exports is at the same pace as the growth of worldwide production. However, the value of fruit and vegetable exports from European countries is steadily declining (Czyżewski and Czakowski 2017). Postharvest losses relate to the degradation in the quantity and quality of the crop's products from harvesting to consumer usage. In many developing countries, like Pakistan, the postharvest loss is a problem of food security and is of concern to everyone. Inappropriate handling of agricultural products after harvest may cause quality and quantity losses. It also accounts for the increasing prices of agricultural products in Pakistan. The total production of vegetables and fruits in Pakistan is nearly 13.764 million tons, and it is estimated that 35%–40% of vegetables and fruits are wasted after harvesting. Postharvest loss is one of the concerns of food security and global hunger in many countries (Ahmada, Khanc, and Sarward 2021).

Major causes of postharvest losses in fruits and vegetables occur during harvesting, postharvest handling and storage, processing stages, distribution, and consumption. Therefore, the use of appropriate postharvest handling, packaging, transportation, and storage practices is very important to minimize the amount of postharvest loss. Due to improved living standards in recent years, fruit and vegetable sales have gradually increased, disseminating from a local scale to a national and even global scale, requiring enhanced storage conditions for fresh produce (Elik et al. 2019). The annual global loss ranges between 30% and 50% (Oluwatayo, Clement, and Bamidele 2019). Fruit and vegetable degradation not only presents a threat to human health but also causes enormous economic losses in the food industry (Thomas and Silke 2014).

Using extracts from fruits, vegetables, or plants in the biological production of nanoparticles (NPs) is an appealing method as they are easily available. Plant extracts having valuable functional groups, including hydroxyl, carbonyl, carboxyl, and phenol groups, contain antioxidants such as flavonoids and phenolic acids, which are used to synthesize nanoparticles. These functional groups play a role in the reduction of different metals, including palladium, gold, and silver, into their corresponding metallic nanoparticles (Ismail et al. 2017). Fruit wastes have been suggested as a good secondary resource for making silver nanoparticles, however, their usage is relatively new (Nasiriboroumand, Montazer, and Barani 2018). Transformation of bio wastes into various value‐added products is also a way of efficiently achieving a circular economy and managing waste (Barros et al. 2018).

Nano science is basically the study of tremendously small things, and it is an interdisciplinary field that includes the chemical, biological, biotechnology, engineering, and material sciences. Nanomaterials often have a larger surface area to volume ratio than bulk materials, which helps with catalysis (Yaqoob, Umar, and Ibrahim 2020). Silver nanoparticles are distinctive in terms of their biological, chemical, and physical properties. As a result, several uses for these nanoparticles have been found. Silver nanoparticles have been synthesized using a variety of techniques. However, the conventional chemical and physical techniques used to synthesize silver nanoparticles appear to be costly and hazardous. Biological silver nanoparticle preparation, on the other hand, appears to be simple, quick, safe, reliable, environmentally friendly, and capable of producing well‐defined size and shape under optimized circumstances, therefore, it appears to address these drawbacks. Moreover, high yield, stability and solubility are produced by biological synthesis (Castillo‐Henríquez et al. 2020).

The application could be used to prolong the shelf life of fruits and vegetables. Because of their enhanced mechanical, thermal, barrier, and antimicrobial qualities, the nanocomposite antimicrobial packaging solutions are promising options (Jafarzadeh et al. 2019). Fruit preservation can be aided by the application of films and coatings. The coating is the thin layer of a substance that acts as a barrier between food and its surroundings (Majid et al. 2018). Functional coatings developed recently not only change the internal environment of fruits but also add value to the product. These coatings let fresh fruits hold onto their physicochemical characteristics and phytochemicals for an extended amount of time. Compared to alternative preservation techniques, coatings also result in cost savings. Thus, it can be said that coating is among the most effective ways to keep fresh fruits fresh (Sharma et al. 2019).

Biosynthesis of nanoparticles, especially silver nanomaterials from plant extracts or organic sources, has been receiving huge interest because of their plentiful abilities and a wide range of bioactive reducing metabolites. Plants are known as highly preferable sources for synthesizing nanoparticles (Zhang et al. 2016). Compared with bacteria and algae, plants are more renitent to metal toxicity, thereby offering a green alternative for synthesis of silver nanoparticles. The biological approach for synthesizing silver nanoparticles is increasingly considered. This method is a green technology aimed at minimizing the negative environmental impact. It has been known that to synthesize silver nanoparticles a using chemical approach, mainly three ingredients are required: a silver salt, a stabilizer or capping agent, and a reducing agent (Gurunathan et al. 2014).

Green chemistry becomes increasingly crucial for environmental sustainability, utilizing the natural properties of phytochemicals in fruit and vegetable peel waste to produce nanoparticles (NPs) with novel attributes. This study explores the green synthesis of silver nanoparticles (AgNPs) from biological waste, leveraging the inherent reducing agents, antioxidants, and flavonoids in these peels. By drying peel waste from apples, tomatoes, carrots, capsicum, and cucumber, we aim to develop AgNPs with enhanced antibacterial properties. The experiment involves dividing the samples into three groups: one receives no treatment, another is exposed to nanoparticles without silver nitrate, and a third is treated with silver nanoparticles. These groups are stored at room temperature for 15 days and assessed for various physiochemical parameters, including pH, total titratable acidity, texture, total soluble solids, color, and visual decay at 0 and 15 days. Additionally, weight loss is measured at 0, 5, 10, and 15 days of storage. Statistical analysis of the data identifies the most effective treatment for extending the shelf life of fruits and vegetables.

## 2 Materials and Methods

### 2.1 Procurement of Raw Material

Seasonal fruits and vegetables (apples, tomatoes, carrots, capsicum, and cucumber) and their peel waste were collected on a home scale. Other chemicals like deionized water, silver nitrate, 0.1 M sodium hydroxide, and phenolphthalein were procured from Sigma‐Aldrich.

### 2.2 Drying of Peels Fruits and Vegetables Waste

Peels of fruits and vegetables were washed with deionized water and then dried in sunlight for 10–15 days. Fruit and vegetable peels were cleaned with deionized water and then allowed to dry for 10–15 days in sunlight. Dried fruit and vegetable peels were powdered and stored in a container for further processing (Tripathi and Sirohi 2016).

### 2.3 Preparation of Fruits and Vegetables Peel Extract

After drying 25 g of peels were cooked for 30 min at 60°C in 100 mL of deionized water. After boiling, the aqueous solution color changes from colorless to light and dark yellow colors. To eliminate heavy biomaterials, the aqueous extracts were separated by filtration through Whatman No. 1 filter paper (pore size 0.45 μm), followed by centrifugation at 1000 rpm for 10 min (Tripathi and Sirohi 2016).

### 2.4 Synthesis of Silver Nanoparticles

The production of silver nanoparticles was carried out using an aqueous solution of 1 mM silver nitrate (AgNO_3_). Ten microliters of peel extract of each sample was combined with 90 mL of an aqueous solution containing 1 mM silver nitrate to facilitate reduction into Ag+. The mixture was then allowed to incubate for 15 h at ambient temperature. One microMolar of AgNO_3_ is reduced and stabilized by the filtrate. A change in color from lighter to darker indicated the development of silver nanoparticles (Tripathi and Sirohi 2016).

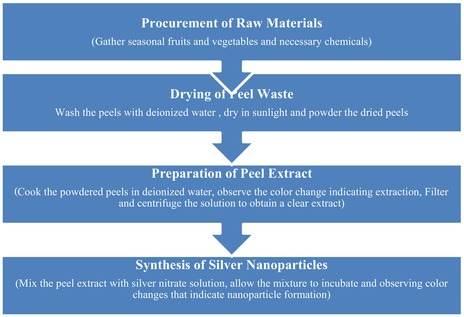

### 2.5 UV–VIS Spectroscopy

The UV–VIS Spectrophotometer V‐530 JASCO was utilized to characterize the silver nanoparticles. The samples were scanned in the 300–800 nm range. As a reference, double‐distilled water was utilized (Tripathi and Sirohi 2016).

### 2.6 Application of Nanoparticles to Fruits and Vegetable

Sample was prepared according to slight modification in the method by Robak, Kotkowiak, and Drozdowski (2015). Five different types of fruits and vegetables were used in this research. Three treatments were designed with different proportions: one was controlled, the other treated with nanoparticles from fruits and vegetable peel, and the last was treated with silver nanoparticles, where T_V0_ is control vegetable, T_VNP_ is vegetable treated with nanoparticles from fruits and vegetables peel, and the third group T_VNSP_ is treated with silver nanoparticles using peel extract. Similarly, as T_F0_ is control fruits, and T_FNP_ is treated with nanoparticles from fruits and vegetables peel waste, and third group T_FSNP_ is treated with fruit treated with silver nanoparticles using peel extract as shown in Table 1.

**TABLE 1:** Treatment plan for fruits (apples and tomatoes) and vegetables (carrots, capsicum, and cucumber) treated with nanoparticles and silver nanaoparticles.

No. of commodities	Control	Nanoparticles from fruits and vegetables peel waste	Silver nanoparticles
Vegetables	T_V0_	T_VNP_	T_VNSP_
Fruits	T_F0_	T_FNP_	T_FSNP_

### 2.7 Storage Study

Fruit and vegetable samples that had been treated and packaged in LLDPE (linear low density polyethylene) boxes and stored for 15 days at ambient temperature in order to be analyzed.

### 2.8 Physiochemical Analysis of Fruits (Apples and Tomatoes) and Vegetables (Carrots, Capsicum, and Cucumber)

Physiochemical analysis of fruits and vegetables (pH, total titratable acidity, weight loss, texture, total soluble solids, and color analysis) was analyzed at 0 and 15th day of storage after applying nanoparticles and silver nanoparticles were analyzed by respective methods.

#### 2.8.1 pH of Fruits and Vegetables

Fruits and vegetables were tested for pH on 0 and 15th day of storage by using a calibrated model pH meter AOAC (2016).

#### 2.8.2 Total Titratable Acidity of Fruits and Vegetables

Titratable acidity of fruits and vegetables was determined at 0 and 15th day by using an acidic meter. Extracted juice from each treatment was taken as the representative sample. Then 30 mL of distilled water was taken in a beaker, 5 mL of sample was adden, shaken well, and then placed on an acidity meter, and the reading was taken directly (Goswami et al. 2015).

#### 2.8.3 Texture Analysis of Fruits and Vegetables

Texture analysis of fruits and vegetables was analyzed at 0 and 15th day and measured according to the method by Sharma et al. (2019). To compress or stretch a sample, a texture analyzer moves in either an up or down direction. A load cell is mounted on the moving arm, which records the force response of the sample to the deformation imparted on it.

Fruits and vegetables were analyzed for texture at 0 and 15th day and measured by the method given by Sharma et al. (2019). To compress or stretch a sample, a texture analyzer moves in either an up or down direction. The moving arm has a load cell installed on it that measures the sample's force response to deformation.

#### 2.8.4 Total Soluble Solids of Fruits and Vegetables

Total soluble solids of fruits and vegetables was analyzed at 0 and 15th day and determined by using a refractometer (Eclips refractometer made in UK) at room temperature and expressed as a percentage by AOAC (2016). The sample was well shaken, and the representative sample was put on the dried prism of the refractometer and then the reading was taken directly.

#### 2.8.5 Color Analysis of Fruits and Vegetables

Color analysis of fruits and vegetables was analyzed at 0 and 15th day and measured according to the method by Filipović (2016). The sample of fruits and vegetables was placed in front of the colorimeter lens and the button was pressed, the values of L*, a*, and b* was noted. The procedure repeated three times to obtain more accuracy. The results were expressed in terms of L* lightness, a* redness to greenness and b* yellowness. The measurement was conducted using a white standard and the same light conditions under artificial fluorescent light at room temperature.

#### 2.8.6 Visual Decay

The visual decay of the fruits and vegetables was analyzed at 0 and 15th day of storage according to the method given by Ali et al. (2020).

#### 2.8.7 Weight Loss of Fruits (Apples and Carrots) and Vegetables (Carrots, Capsicum, and Cucumber)

The weight loss was examined at Day 0, 5, 10, and 15. Before storage, three fruits and vegetables from each treatment were labeled and weighed using a weighing balance by Qin et al. (2015). At the beginning of the experiment, and at the end of each storage cycle, the same fruits were weighed. Following equation is used to calculate weight loss:

$$\%\text{Weight loss}=\frac{M_{0}-M_{1}}{M_{0}}\times 100$$

where *M*_0_ is the initial weight and *M*_1_ is the final weight.

## 3 Results and Discussion

The reported attribute and their corresponding finding are deliberated and discussed herein.

### 3.1 Scanning Electron Microscopic (SEM) Studies of Nanoparticles Prepared From Fruits and Vegetables Peel Waste

**FIGURE 1:**
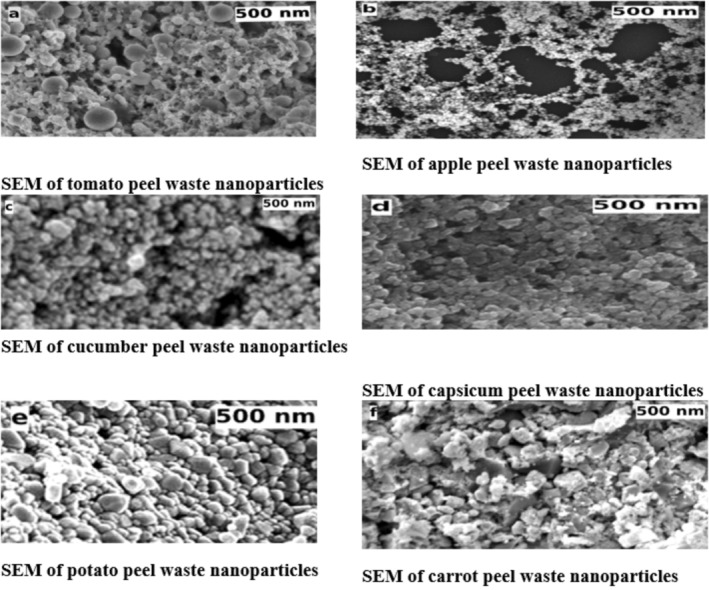
Scanning electron microscope (SEM) studies of vegetables and fruit peel waste nanoparticles.

SEM analysis was used to examine the morphology of nanoparticles prepared from fruits and vegetables peel waste. SEM study of nanoparticles at 500 nm offers important information about their size distribution, surface appearance, and structural homogeneity as illustrated in Figure 1. The high spatial resolution provided by SEM enables researchers to quantify particle dimensions and evaluate size uniformity, which is essential for meeting application‐specific requirements (Reddy 2017).

### 3.2 Physiochemical Analysis of Fruits (Apples and Tomatoes) and Vegetables (Carrots, Capsicum, and Cucumber)

#### 3.2.1 pH Analysis of Fruits and Vegetables

##### 3.2.1.1 pH Analysis of Apples

Mean values for pH of apples are exposed in Table 2. Triplicate samples of fruits were used for each treatment, and pH was analyzed at 0 day (fresh) and at 15th day of storage. At 0 day, results showed that the pH value of T_F0_ (control) expressed 3.35 ± 0.04. Whereas treatments T_FNP_ (fruits with nanoparticles) and T_FSNP_ (fruits with silver nanoparticles) expressed 3.38 ± 0.04 and 3.42 ± 0.02 pH of apples, respectively. At 15th day of storage, results exhibited that the pH of apples in T_F0_ showed 3.30 ± 0.02, whereas treatments T_FNP_ and T_FSNP_ expressed 3.34 ± 0.06 and 3.39 ± 0.00 pH of apples, respectively. The current pH results are consistent with those of Azmat et al. (2017), who found that the pH of apples ranged from 3.25 to 3.43. During the storage time period (0 and 15 day) T_F0_ acquired highest pH value on 15th day of storage interval, so the increase in pH value during storage was due to a decrease in titratable acidity which decreased the amount of malic acid in apples and treatments T_FNP_ and T_FSNP_ showed almost maintained the pH of apples because nanoparticles and silver nanoparticles preserve the pH of apples as fresh during storage, so these two treatments indicate the best storage of apples with better shelf life. During this study, with respect to treatments, the pH of apples ranged from 3.00 to 3.15. However, in terms of days, the pH of apples was 3.19 at day 0 and declined to 3.06 at day 15.

**TABLE 2:** Effect of treatments and days on pH of fruits and vegetables.

Treatment	Apples			Tomatoes			Carrots			Capsicum			Cucumber		
	0	15	Mean	0	15	Mean	0	15	Mean	0	15	Mean	0	15	Mean
T_F0_	3.35 ± 0.04c	3.30 ± 0.02a	3.00a	3.13 ± 0.2d	3.08 ± 0.01a	3.00a	5.90 ± 0.01b	5.8 ± 0.04a	6.03a	4.55 ± 0.01cd	4.50 ± 0.06a	4.22a	3.18 ± 0.02c	3.22 ± 0.01a	3.60a
T_FNP_	3.38 ± 0.04c	3.34 ± 0.06a	3.10a	3.15 ± 0.02d	3.10 ± 0.08b	3.05b	5.95 ± 0.01c	5.93 ± 0.01b	6.05b	4.58 ± 0.02d	4.56 ± 0.01b	4.25b	3.22 ± 0.01d	3.1 ± 0.02b	3.63b
T_FSNP_	3.42 ± 0.03c	3.39 ± 0.00b	3.15a	3.17 ± 0.01d	3.15 ± 0.01c	3.09b	6.00 ± 0.02d	5.98 ± 0.01c	6.07c	4.60 ± 0.03d	4.57 ± 0.01bc	4.29b	3.24 ± 0.01f	3.22 ± 0.01c	3.65c
Mean	3.19b	3.06a		3.20a	3.06b		6.20a	6.15a		4.30a	4.25a		3.04a	3.00b	

Abbreviations: T_V0_ = vegetables control; T_VNP_ = vegetables with nanoparticle; T_VNSP_ = vegetables with silver nanoparticle.

##### 3.2.1.2 pH Analysis of Tomatoes

Mean values for pH of tomatoes are shown in Table 2. Triplicate samples of fruits were used for each treatment, and pH was analyzed at 0 day (fresh) and at 15th day of storage. At 0 day, results showed that the pH value of T_F0_ (control) expressed 3.13 ± 0.2, whereas treatments T_FNP_ (fruit with nanoparticles) and T_FSNP_ (fruit with silver nanoparticles) expressed 3.15 ± 0.04 and 3.17 ± 0.01. pH of tomatoes, respectively. According to recommendations, industrial tomatoes should have a pH between 3.18 and 4.4 (Aboagye‐Nuamah, Hussein, and Ackun 2018). At 15th day of storage, results exhibited that the pH of tomatoes in T_F0_ showed 3.08 ± 0.01, whereas treatments T_FNP_ and T_FSNP_ expressed 3.10 ± 0.08 and 3.15 ± 0.01 pH of tomatoes, respectively. During the storage time period (0 and 15 days), T_F0_ acquired the highest pH value on 15th day of storage interval because temperature and storage time affect tomatoes pH as well its shelf life. Treatments T_FNP_ and T_FSNP_ showed almost maintained the pH of tomatoes because nanoparticles and silver nanoparticles preserve the pH of tomatoes as fresh during storage, so these two treatments indicate the best storage of tomatoes with better shelf life. Tomato pH varied during this study, ranging from 3.00 to 3.09 depending on the treatments. But in terms of days, the tomatoes' pH was 3.20 on day 0 and dropped to 3.06 by day 15.

##### 3.2.1.3 pH Analysis of Carrots

Triplicate samples of vegetables were used for each treatment, and pH was analyzed at 0 day (fresh) and at 15th day of storage. At 0 day, results showed that pH value of T_V0_ (control) expressed 5.90 ± 0.01, whereas treatments T_VNP_ (vegetable with nanoparticles) and T_VSNP_ (vegetable with silver nanoparticle) expressed 5.95 ± 0.01 and 6.00 ± 0.02 pH of carrots, respectively. At 15th day of storage, results exhibited that the pH of carrots in T_V0_ shows 5.85 ± 0.04, whereas treatments T_VNP_ and T_VNSP_ expressed 5.93 ± 0.01 and 5.98 ± 0.01 pH of carrots, respectively. The current results are conform to the outcomes of Martínez‐Hernández, Amodio, and Colelli (2017) who reported that the pH of carrots ranges from 5.57 to 6.10. During storage (0 and 15 day), T_V0_ acquired the highest pH value at the 15th day of storage interval, and treatments T_VNP_ and T_VNSP_ showed almost maintained the pH of carrots because nanoparticles and silver nanoparticles preserve the pH of carrots as fresh during storage, so these two treatments indicate the best storage of carrots with better shelf life. During this study, with respect to treatments, the pH of carrots ranged from 6.03 to 6.07. However, with respect to days, the pH of carrots was 6.20 at day 0 and dropped to 6.15 at day 15.

##### 3.2.1.4 pH Analysis of Capsicum

Mean values for pH of capsicum are exposed in Table 2. Triplicates samples of vegetable were used for each treatment, and pH analyzed at 0 day (fresh) and at 15th day of storage. At 0 day results showed that pH value of T_V0_ (control) expressed 4.55 ± 0.01. Whereas treatments T_VNP_ (vegetable with nanoparticles) and T_VNSP_ (vegetable with silver nanoparticles) expressed 4.58 ± 0.02 and 4.60 ± 0.03 pH of capsicum respectively. At 15th day of storage result exhibited that the pH of capsicum in T_V0_ showed 4.50 ± 0.06. Whereas treatments T_VNP_ and T_VNSP_ expressed 4.56 ± 0.01 and 4.57 ± 0.01 pH of capsicum respectively. Similarly, Gonzalo et al. (2020) evaluated that the pH of capsicum ranged from 4.60 to 5.50. During storage period (0 and 15 day) T_V0_ acquired highest pH value on 15th day of storage interval and treatments T_VNP_ and T_VNSP_ showed almost maintained the pH of capsicum because nanoparticles and silver nanoparticles preserves the pH of capsicum as fresh during storage so these two treatments indicate the best storage of capsicum with better shelf life. During this study, with respect to treatments pH of capsicum ranged from 4.22 to 4.29. However, with respect to days, pH of capsicum is 4.60 at 0 day and decreased to 4.57 at 15th day.

##### 3.2.1.5 pH Analysis of Cucumber

Mean values for pH of cucumber are exposed in Table 2. Triplicate samples of vegetable were used for each treatment, and pH was analyzed at 0 day (fresh) and at 15th day of storage. At 0 day, results showed that the pH value of T_V0_ (control) expressed 3.18 ± 0.02, whereas treatments T_VNP_ (vegetables with nanoparticles) and T_VNSP_ (vegetables with silver nanoparticles) expressed 3.22 ± 0.01 and 3.25 ± 0.01 pH of cucumber, respectively. At 15th day of storage, results exhibited that the pH of capsicum in T_V0_ showed 3.22 ± 0.01, whereas treatments T_VNP_ and T_VNSP_ expressed 3.18 ± 0.02 and 3.22 ± 0.01 pH of cucumber, respectively. Similarly, Sidonia et al. (2007) evaluated that the pH of cucumber ranged from 3.20 to 3.50. During the storage time period (0 and 15 day), T_V0_ acquired the highest pH value on 15th day of storage interval, and treatments T_VNP_ and T_VNSP_ showed that they almost maintained the pH of cucumber because nanoparticles and silver nanoparticles preserves the pH of cucumber as fresh during storage so these two treatments indicate the best storage of cucumber with better shelf life. During this study, with respect to treatments, the pH of cucumber ranged from 3.60 to 3.65. However, with respect to days, the pH of cucumber is 3.04 at 0 day and decreased to 3.00 at 15th day.

#### 3.2.2 Titratable Acidity Analysis of Fruits (Apples and Tomatoes) and Vegetables (Carrots, Capsicum and Cucumber)

##### 3.2.2.1 Titratable Acidity of Apples

Mean values for the titratable acidity value of apples are shown in Table 3. Triplicate samples of fruits were used for each treatment, and titratable acidity analyzed at 0 day (fresh) and at 15th day of storage. At 0 day, results showed that the titratable acidity value of T_F0_ (control) expressed 1.15 ± 0.03 g/L, whereas treatments T_FNP_ (fruit with nanoparticle) and T_FSNP_ (fruit with silver nanoparticle) expressed 1.18 ± 0.01 and 1.21 ± 0.01 g/L titratable acidity values of apples, respectively. At 15th day of storage, result exhibited that the titratable acidity analysis of apples in T_F0_ showed 1.17 ± 0.02 g/L, whereas treatments T_FNP_ and T_FSNP_ expressed 1.20 ± 0.03 and 1.23 ± 0.01 g/L titratable acidity values of apples, respectively. The current results of titratable acidity are in accordance with the findings of Azmat et al. (2017), who observed that titratable acidity of apples was ranged from 1.20 to 1.38. During the storage time period (0 and 15 day), T_F0_ acquired a great decrease in titratable acidity value on 15th day of storage interval, and treatments T_FNP_ and T_FSNP_ showed almost maintained the titratable acidity values of apples because nanoparticles and silver nanoparticles preserve the titratable acidity values of apples as fresh during storage, so these two treatments indicate the best storage of apples with better shelf life. During this study, with respect to treatments, the titratable acidity value of apples ranged from 1.40 to 1.43 g/L. However, with respect to days, titratable acidity value of apples was found to be 1.45 g/L at 0 day and increased at 15th day, which was 1.47 g/L. The increase in acidity of apples might be due to the addition of citric and ascorbic acid and also due to the break down of sugar into acids during dehydration and storage.

**TABLE 3:** Effect of treatments and days on titratable acidity (g/L) of fruits and vegetables.

Treatment	Apples			Tomatoes			Carrots			Capsicum			Cucumber		
	0	15	Mean	0	15	Mean	0	15	Mean	0	15	Mean	0	15	Mean
T_F0_	1.15 ± 0.03a	1.17 ± 0.02d	1.40b	0.63 ± 0.01a	0.60 ± 0.02b	0.39b	0.30 ± 0.01a	0.15 ± 0.04d	0.29c	0.33 ± 0.02a	0.24 ± 0.01b	0.28b	0.33 ± 0.01a	0.30 ± 0.01d	0.40b
T_FNP_	1.18 ± 0.01b	1.20 ± 0.03d	1.42b	0.67 ± 0.01b	0.66 ± 0.04c	0.42b	0.34 ± 0.01a	0.31 ± 0.01c	0.32b	0.36 ± 0.04b	0.34 ± 0.02a	0.30b	0.35 ± 0.02b	0.32 ± 0.02e	0.43b
T_FSNP_	1.21 ± 0.01a	1.23 ± 0.01c	1.43a	0.72 ± 0.02c	0.70 ± 0.03d	0.44a	0.36 ± 0.01a	0.34 ± 0.01b	0.34a	0.39 ± 0.02c	0.36 ± 0.03b	0.32a	0.37 ± 0.01c	0.34 ± 0.01f	0.46a
Mean	1.45a	1.47b		0.54a	0.44b		0.38a	0.32b		0.37a	0.27b		0.23a	0.17b	

Abbreviations: T_V0_ = vegetables control; T_VNP_ = vegetables with nanoparticle; T_VNSP_ = vegetables with silver nanoparticle.

##### 3.2.2.2 Titratable Acidity of Tomatoes

Mean values for the titratable acidity value of tomatoes are explained in Table 3. Triplicate samples of fruits were used for each treatment, and titratable acidity was analyzed at 0 day (fresh) and at 15th day of storage. At 0 day, results showed that the titratable acidity value of T_F0_ (control) expressed 0.63 ± 0.01 g/L, whereas treatments T_FNP_ (fruits with nanoparticles) and T_FSNP_ (fruits with silver nanoparticles) expressed 0.67 ± 0.01 and 0.672 ± 0.02 g/L titratable acidity values of tomatoes, respectively. At 15th day of storage, results exhibited that the titratable acidity analysis of tomatoes in T_F0_ showed 0.60 ± 0.02 g/L, whereas treatments T_FNP_ and T_FSNP_ expressed 0.66 ± 0.04 and 0.70 ± 0.03 g/L titratable acidity values of tomatoes, respectively. In a research trail conducted by Gyanendra et al. (2012), they reported that the titratable acidity of tomatoes ranged from 0.63 to 0.72 g/L. During the storage time period (0 and 15 day), T_F0_ acquired higher decrease in titratable acidity value on 15th day of storage interval, and treatments T_FNP_ and T_FSNP_ showed almost maintained the titratable acidity values of tomatoes because nanoparticles and silver nanoparticles preserve the titratable acidity values of tomatoes as fresh during storage, so these two treatments indicate the best storage of tomatoes with better shelf life. During this study, with respect to treatments, the titratable acidity value of tomatoes ranged from 0.39 to 0.44 g/L. However, with respect to days, the titratable acidity value of tomatoes was 0.54 at 0 day and decreased to 0.44 g/L at 15th day.

##### 3.2.2.3 Titratable Acidity of Carrots

Mean values for the titratable acidity value of carrots are exposed in Table 3. Triplicate samples of vegetable were used for each treatment, and titratable acidity was analyzed at 0 day (fresh) and at 15th day of storage. At 0 day, results showed that the titratable acidity value of T_V0_ (control) expressed 0.30 ± 0.01 g/L, whereas treatments T_VNP_ (vegetable with nanoparticle) and T_VNSP_ (vegetable with silver nanoparticle) expressed 0.34 ± 0.01 and 0.36 ± 0.01 g/L titratable acidity values of carrots, respectively. At 15th day of storage, results exhibited that the titratable acidity analysis of carrots in T_V0_ showed 0.15 ± 0.04 g/L, whereas treatments T_VNP_ and T_VNSP_ expressed 0.31 ± 0.01 and 0.34 ± 0.01 g/L titratable acidity values of carrots, respectively. However, the results of present study are supported by the previous findings of Ali, Nurasyikin, and Maysoun (2015) in which titratable acidity of carrots ranged from 0.37 to 0.49 g/L. During the storage time period (0 and 15 day), T_V0_ acquired a higher decrease in titratable acidity value on 15th day of storage interval, and treatments T_VNP_ and T_VNSP_ showed almost maintained the titratable acidity values of carrots because nanoparticles and silver nanoparticles preserve the titratable acidity values of carrots as fresh during storage, so these two treatments indicate the best storage of carrots with better shelf life. During this study, with respect to treatments, the titratable acidity value of carrots ranged from 0.29 to 0.34 g/L. However, with respect to days, the treatable acidity value of carrots was 0.38 g/L at 0 day and decreased to 0.32 g/L at 15th day.

##### 3.2.2.4 Titratable Acidity of Capsicum

Mean values for the titratable acidity value of capsicum are shown in Table 3. Triplicate samples of vegetables were used for each treatment, and titratable acidity was analyzed at 0 day (fresh) and at 15th day of storage. At 0 day, results showed that titratable acidity value of T_V0_ (control) expressed 0.33 ± 0.02 g/L, whereas treatments T_VNP_ (vegetable with nanoparticles) and T_VNSP_ (vegetable with silver nanoparticles) expressed 0.36 ± 0.04 and 0.39 ± 0.02 g/L titratable acidity values of capsicum, respectively. At 15th day of storage, results exhibited that the titratable acidity analysis of capsicum in T_V0_ showed 0.24 ± 0.01 g/L, whereas treatments T_VNP_ and T_VNSP_ expressed 0.34 ± 0.02 and 0.36 ± 0.03 g/L titratable acidity values of capsicum, respectively. Similarly, Satish, Thakur, and Kiran (2019) evaluated that the titratable acidity of capsicum ranged from 0.25 to 0.37 g/L. During the storage time period (day 0 and 15), T_V0_ acquired higher decrease in titratable acidity value on 15th day of storage interval, and treatments T_VNP_ and T_VNSP_ showed almost maintained the titratable acidity values of capsicum because nanoparticles and silver nanoparticles preserve the titratable acidity values of capsicum as fresh during storage, so these two treatments indicate the best storage of capsicum with better shelf life. During this study, with respect to treatments, titratable acidity value of capsicum ranged from 0.28 to 0.32 g/L. However, with respect to days, titratable acidity value of capsicum was 0.37 g/L at 0 day and decreased to 0.27 g/L at 15th day.

##### 3.2.2.5 Titratable Acidity of Cucumber

Mean values for the titratable acidity value of cucumber are exposed in Table 3. Triplicate samples of vegetable were used for each treatment, and titratable acidity was analyzed at 0 day (fresh) and at 15th day of storage. At 0 day, results showed that titratable acidity value of T_V0_ (control) expressed 0.33 ± 0.01 g/L, whereas treatments T_VNP_ (vegetables with nanoparticles) and T_VNSP_ (vegetables with silver nanoparticles) expressed 0.35 ± 0.02 and 0.37 ± 0.01 g/L titratable acidity values of cucumber respectively. At 15th day of storage result exhibited that the titratable acidity analysis of cucumber in T_V0_ showed 0.30 ± 0.01 g/L, whereas treatments T_VNP_ and T_VNSP_ expressed 0.32 ± 0.02 and 0.34 ± 0.01 g/L titratable acidity values of cucumber respectively. During the storage time period (0 and 15 day) T_V0_ acquired higher decrease in titratable acidity value on 15th day of storage interval, and treatments T_VNP_ and T_VNSP_ showed almost maintained the titratable acidity values of cucumber because nanoparticles and silver nanoparticles preserve the titratable acidity values of cucumber as fresh during storage so these two treatments indicate the best storage of cucumber with better shelf life. Similarly, Olufunmilayo and Uzoma (2016) evaluated that the pH of cucumber ranged from 0.21 to 0.36 g/L. During this study, with respect to treatments, the titratable acidity value of cucumber ranged from 0.40 to 0.46 g/L. However, with respect to days, the titratable acidity value of cucumber was 0.23 g/L at 0 day and decreased to 0.17 g/L at 15th day.

#### 3.2.3 Texture Analysis of Fruits (Apples and Tomatoes) and Vegetables (Carrots, Capsicum, and Cucumber)

##### 3.2.3.1 Texture Analysis of Apples

Mean values for the texture of apples are exposed in Table 4. Triplicate samples of fruits were used for each treatment, and texture was analyzed at 0 day (fresh) and at 15th day of storage. At 0 day, results showed that the texture value of T_F0_ (control) expressed 25.80 ± 0.01 N, whereas treatments T_FNP_ (fruits with nanoparticles) and T_FSNP_ (fruits with silver nanoparticles) expressed 27.45 ± 0.02 and 30.00 ± 0.01 N texture of apples, respectively. At 15th day of storage, the results exhibited that the texture of apples in T_F0_ showed 23.13 ± 0.01 N, whereas treatments T_FNP_ and T_FSNP_ expressed 25.07 ± 0.04 N and 28.85 ± 0.01 N texture of apples, respectively. The current outcomes are also in conformity with the observations expressed that the texture of apples was 30.03 N, recorded by Valérie, Sara, and Pual (2018). During the storage time period (0 and 15 day), T_F0_ acquired a highly decreased texture value on 15th day of storage interval, and treatments T_FNP_ and T_FSNP_ showed almost maintained the texture of apples because nanoparticles and silver nanoparticles preserve the texture of apples as fresh during storage, so these two treatments indicate the best storage of apples with better shelf life. During this study, with respect to treatments, the texture value of apples ranged from 22.46 to 26.23 N. However, with respect to days, the texture of apples was 29.28 N at 0 day and decreased to 24.02 N at 15th day.

**TABLE 4:** Effect of treatments and days on texture (*N*) of fruits and vegetables.

Treatment	Apples			Tomatoes			Carrots			Capsicum			Cucumber		
	0	15	Mean	0	15	Mean	0	15	Mean	0	15	Mean	0	15	Mean
T_F0_	25.80 ± 0.01c	23.13 ± 0.01f	22.46c	6.93 ± 0.54b	6.90 ± 0.03b	6.19a	20.44 ± 0.04c	19.06 ± 0.04f	22.74c	9.09 **±** 0.04a	9.06 ± 0.03c	10.24c	3.00 ± 0.02c	2.98 ± 0.02f	3.44c
T_FNP_	27.45 ± 0.02b	25.07 ± 0.04c	24.26b	6.96 ± 0.31a	6.94 ± 0.02d	6.25c	22.29 ± 0.04b	18.13 ± 0.02c	24.21b	10.13 ± 0.04b	9.08 ± 0.04d	11.35b	3.09 ± 0.03b	2.96 ± 0.01e	3.64b
T_FSNP_	30.00 ± 0.01a	28.85 ± 0.01d	26.23a	7.05 ± 0.32ab	6.92 ± 0.01c	6.30b	24.78 ± 0.02a	20.09 ± 0.02f	28.43a	10.15 ± 0.02a	9.13 ± 0.02c	11.47a	3.15 ± 0.02a	3.05 ± 0.01d	3.68a
Mean	29.28a	24.02b		6.79a	6.69b		23.01 ± 0.02a	21.08 ± 0.02g		18.08a	14.09b		3.19a	3.13b	

Abbreviations: T_V0_ = vegetables control; T_VNP_ = vegetables with nanoparticle; T_VNSP_ = vegetables with silver nanoparticle.

##### 3.2.3.2 Texture Analysis of Tomatoes

Mean values for the texture of tomatoes are given in Table 4. Triplicate samples of fruits were used for each treatment, and texture was analyzed at 0 day (fresh) and at 15th day of storage. At 0 day, the results showed that the texture value of T_F0_ (control) expressed 6.93 ± 0.04 N, whereas treatments T_FNP_ (fruit with nanoparticles) and T_FSNP_ (fruit with silver nanoparticles) expressed 6.96 ± 0.31 N and 7.05 ± 0.02 N texture of tomatoes, respectively. At 15th day of storage, results exhibited that the texture of tomatoes in T_F0_ showed 6.90 ± 0.03 N, whereas treatments T_FNP_ and T_FSNP_ expressed 6.94 ± 0.02 and 6.92 ± 0.01 N texture of tomatoes, respectively. The current outcomes are also in conformity with the observations expressed that the texture of tomatoes was 7.09 N, recorded by Valérie, Sara, and Pual (2018). During the storage time period (0 and 15 day), T_F0_ acquired a highly decreased texture value on 15th day of storage interval, and treatments T_FNP_ and T_FSNP_ showed almost maintained the texture of tomatoes because nanoparticles and silver nanoparticles preserve the texture of tomatoes as fresh during storage. Thus, these two treatments indicate the best storage of tomatoes with better shelf life. During this study, with respect to treatments, the texture value of tomatoes ranged from 6.19 to 6.30 N. However, with respect to days, the texture of tomatoes was 6.79 at 0 day and decreased to 6.69 N at 15th day.

##### 3.2.3.3 Texture Analysis of Carrots

Mean values for the texture of carrots are presented in Table 4. Triplicate samples of vegetables were used for each treatment, and texture was analyzed at 0 day (fresh) and at 15th day of storage. At 0 day, results showed that the texture value of T_V0_ (control) expressed 20.44 ± 0.04 N, whereas treatments T_VNP_ (vegetables with nanoparticles) and T_VSNP_ (vegetables with silver nanoparticles) expressed 22.29 ± 0.04 N and 24.78 ± 0.02 N texture of carrots, respectively. At 15th day of storage, results exhibited that the texture of carrots in T_V0_ showed 19.06 ± 0.04 N, whereas treatments T_VNP_ and T_VNSP_ expressed 18.13 ± 0.02 N and 20.09 ± 0.02 N texture of carrots, respectively. Similarly, Ibukunoluwa, Olawuyi, and Wonyoung (2019) evaluated that the texture of carrots ranged from 19.65 to 20.96 N. During the storage time period (0 and 15 day), T_V0_ acquired highly decreased texture value on 15th day of storage interval, while treatments T_VNP_ and T_VNSP_ showed almost maintained the texture of carrots because nanoparticles and silver nanoparticles preserve the texture of carrots as fresh during storage, thus, these two treatments indicate the best storage of carrots with better shelf life. During this study, with respect to treatments, the texture value of carrots ranged from 22.74 to 28.43 N. However, with respect to days, the texture of tomatoes was 30.16 N at 0 day and decreased to 20.09 N at 15th day.

##### 3.2.3.4 Texture Analysis of Capsicum

Mean values for texture of capsicum are explained in Table 4. Triplicate samples of vegetables were used for each treatment, and texture analyzed at 0 day (fresh) and at 15th day of storage. At 0 day, results showed that texture value of T_V0_ (control) expressed 15.42 **±** 0.04 N, whereas treatments T_VNP_ (vegetables with nanoparticles) and T_VSNP_ (vegetables with silver nanoparticles) expressed 17.44 ± 0.04 N and 19.40 ± 0.02 N texture of capsicum, respectively. At 15th day of storage, the result exhibited that the texture of carrots in T_V0_ showed 10.06 ± 0.03 N, whereas treatments T_VNP_ and T_VNSP_ expressed 15.08 ± 0.04 N and 17.13 ± 0.02 N texture of capsicum respectively. Similarly, Cardoso, Demiate, and Danesi (2017) evaluated that the pH of capsicum ranged from 8.50 to 9.19 N. During the storage time period (0 and 15 day), T_V0_ acquired a highly decreased texture value on 15th day of storage interval, and treatments T_VNP_ and T_VNSP_ showed almost maintained the texture of capsicum because nanoparticles and silver nanoparticles preserve the texture of capsicum as fresh during storage, thus, these two treatments indicate the best storage of capsicum with better shelf life. During this study, with respect to treatments, the texture value of capsicum ranged from 18.24 to 16.27 N. However, with respect to days, texture of capsicum was 18.08 N at 0 day and decreased to 12.09 N at 15th day.

##### 3.2.3.5 Texture Analysis of Cucumber

Mean values for the texture of cucumber are shown in Table 4. Triplicate samples of vegetables were used for each treatment, and texture was analyzed at 0 day (fresh) and at 15th day of storage. At 0 day, results showed that the texture value of T_V0_ (control) expressed 3.00 ± 0.02 N, whereas treatments T_VNP_ (vegetables with nanoparticles) and T_VSNP_ (vegetables with silver nanoparticles) expressed 3.08 ± 0.03 N and 3.15 ± 0.02 N texture of cucumber, respectively. However, the results of present study are supported by the previous findings which expressed texture a score of cucumber 3.13 by Nyorere and Uguru (2018). At 15th day of storage, results exhibited that the texture of carrots in T_V0_ showed 2.98 ± 0.02, whereas treatments T_VNP_ and T_VNSP_ expressed 2.96 ± 0.01 N and 3.05 ± 0.01 N texture of cucumber, respectively. During the storage time period (0 and 15 day) T_V0_ acquired a highly decreased texture value on 15th day of storage interval, and treatments T_VNP_ and T_VNSP_ showed almost maintained the texture of cucumber because nanoparticles and silver nanoparticles preserve the texture of cucumber as fresh during storage, so these two treatments indicate the best storage of cucumber with better shelf life. During this study, with respect to treatments, the texture value of capsicum ranged from 3.44 to 3.68 N. However, with respect to days, texture of cucumber is 3.19 N at 0 day and decreased at 15th day which was 3.13 N.

#### 3.2.4 Total Soluble Solids of Fruits (Apples and Tomatoes) and Vegetables (Carrots, Capsicum, and Cucumber)

##### 3.2.4.1 Total Soluble Solids of Apples

Mean values for total soluble solids of apples are exposed in Table 5. Triplicate samples of fruits were used for each treatment, and total soluble solid was analyzed at 0 day (fresh) and at 15th day of storage. At 0 day, results show that the total soluble solid value of T_F0_ (control) expressed 18.32 ± 0.01 Brix, whereas treatments T_FNP_ (fruits with nanoparticles) and T_FSNP_ (fruits with silver nanoparticles) expressed 20.13 ± 0.01 Brix and 22.09 ± 0.01 Brix total soluble solids of apples respectively. At 15th day of storage, results exhibited that the total soluble solids of apples in T_F0_ showed 15.65 ± 0.02 Brix, whereas treatments T_FNP_ and T_FSNP_ expressed 18.16 ± 0.01 Brix and 20.66 ± 0.01 Brix total soluble solids of apples respectively. The current results are interrelated with the previous findings of Zarmeena et al. (2017), they reported that the total soluble solid contents of apples has 20.03–28.03. During the storage time period (day 0 and 15), T_F0_ acquired extremely decrease in total soluble solid values on 15th day of storage interval and treatments T_FNP_ and T_FSNP_ showed minor decrease in the total soluble solids of apples because nanoparticles and silver nanoparticles preserve the total soluble solids of apples as fresh during storage so these two treatments indicate the best storage of apples with better shelf life. Similarly, in a previous study, the treatments with nanoparticles maintained the total soluble solids of apples throughout the storage period (Yang, Li, and Li 2010). During this study, with respect to treatments total soluble solids of apples ranged from 12.99 to 16.88 Brix. However, with respect to days, total soluble solids of apples are 15.18 Brix at 0 day and the value decreased at 15th day to 13.49 Brix.

**TABLE 5:** Effect of treatments and days on total soluble solid (Brix) of fruits and vegetables.

Treatment	Apples			Tomatoes			Carrots			Capsicum			Cucumber		
	0	15	Mean	0	15	Mean	0	15	Mean	0	15	Mean	0	15	Mean
T_F0_	18.32 ± 0.01c	15.65 ± 0.02a	12.99a	12.32 ± 0.04a	7.65 ± 0.03	10.39b	6.0 ± 0.01	5.73 ± 0.02b	6.70a	4.85 ± 0.01	2.70 ± 0.01	3.64a	2.90 ± 0.02d	2.67 ± 0.01a	3.10a
T_FNP_	20.13 ± 0.01d	18.16 ± 0.01b	14.65c	14.13 ± 0.03b	12.16 ± 0.01b	11.00b	6.2 ± 0.01e	6.16 ± 0.04a	6.73b	5.00 ± 0.01b	4.27 ± 0.01b	4.58b	2.95 ± 0.01e	2.90 ± 0.01b	3.23b
T_FSNP_	22.09 ± 0.01e	20.66 ± 0.01b	16.88b	16.09 ± 0.01c	15.66 ± 0.02a	12.05a	6.3 ± 0.01f	6.28 ± 0.03c	6.76c	6.65 ± 0.01a	5.50 ± 0.01c	5.26c	3.00 ± 0.01f	2.95 ± 0.01c	3.25c
Mean	15.18b	13.49a		10.18a	7.00b		6.78	6.70a		5.00a	3.05b		13.00a	11.56b	

Abbreviations: T_V0_ = vegetables control; T_VNP_ = vegetables with nanoparticle; T_VNSP_ = vegetables with silver nanoparticle.

##### 3.2.4.2 Total Soluble Solids of Tomatoes

Mean values for total soluble solids of tomatoes are shown in Table 5. Triplicate samples of fruits were used for each treatment, and total soluble solid was analyzed at 0 day (fresh) and at 15th day of storage. At 0 day, results showed that the total soluble solid value of T_F0_ (control) expressed 12.32 ± 0.04 Brix, whereas treatments T_FNP_ (fruits with nanoparticles) and T_FSNP_ (fruits with silver nanoparticles) expressed 12.16 ± 0.01 Brix and 15.66 ± 0.02 Brix total soluble solids of tomatoes, respectively. According to a previous study, it has been established that highest amount of total soluble solids is an indication of the highest quality of industrial tomato (Aboagye‐Nuamah, Hussein, and Ackun 2018). At 15th day of storage result exhibited that the total soluble solids of tomatoes in T_F0_ showed 7.65 ± 0.03 Brix. Whereas treatments T_FNP_ and T_FSNP_ expressed 12.16 ± 0.01 Brix and 15.66 ± 0.01 Brix total soluble solids of tomatoes respectively. During the storage time period (0 and 15 day), T_F0_ acquired a highly decrease in total soluble solid value on 15th day of storage interval, and treatments T_FNP_ and T_FSNP_ showed minor decreases in the total soluble solids of tomatoes because nanoparticles and silver nanoparticles preserve the total soluble solids of tomatoes as fresh during storage, so these two treatments indicate the best storage of tomatoes with better shelf life. During this study, with respect to treatments, total soluble solids of tomatoes ranged from 10.39 to12.05 Brix. However, with respect to days, total soluble solids of tomatoes is 10.18 at 0 day and the value decreases at 15th day to 7.00 Brix.

##### 3.2.4.3 Total Soluble Solids of Carrots

Mean values for total soluble solids of carrots are exposed in Table 5. Triplicate samples of vegetables were used for each treatment, and total soluble solids were analyzed at 0 day (fresh) and at 15th day of storage. At 0 day, results showed that total soluble solids value of T_V0_ (control) expressed 0.75 ± 0.01 Brix, whereas treatments T_VNP_ (vegetables with nanoparticles) and T_VNSP_ (vegetables with silver nanoparticles) expressed 0.78 ± 0.01 Brix and 0.80 ± 0.01 Brix total soluble solids of carrots, respectively. At 15th day of storage, results exhibited that the total soluble solids of carrots in T_V0_ showed 0.73 ± 0.02 Brix, whereas treatments T_VNP_ and T_VNSP_ expressed 0.76 ± 0.04 Brix and 0.79 ± 0.03 Brix total soluble solids of carrots, respectively. Similarly, Ali, Nurasyikin, and Maysoun (2015) evaluated that the total soluble solid contents of carrots ranged from 0.81 to 1.00 Brix. During the storage period (0 and 15 day), T_V0_ acquired a highly decrease in total soluble solids value on 15th day of storage interval, and treatments T_VNP_ and T_VNSP_ showed minor decrease in the total soluble solids of carrots because nanoparticles and silver nanoparticles preserve the total soluble solids of carrots as fresh during storage, so these two treatments indicate the best storage of carrots with better shelf life. During this study, with respect to treatments, total soluble solids of carrots ranged from 0.70 to 0.76 Brix. However, with respect to days, total soluble solids of carrots is 0.78 Brix at 0 day and the value decreases at 15th day to 0.70 Brix.

##### 3.2.4.4 Total Soluble Solids of Capsicum

Mean values fortotal soluble solids of capsicum are exposed in Table 5. Triplicate samples of vegetables were used for each treatment, and total soluble solid was analyzed at 0 day (fresh) and at 15th day of storage. At 0 day, results showed that the total soluble solid value of T_V0_ (control) expressed 6.00 ± 0.01 Brix, whereas treatments T_VNP_ (vegetables with nanoparticles) and T_VNSP_ (vegetables with silver nanoparticles) expressed 6.25 ± 0.01 Brix and 6.30 ± 0.01 Brix total soluble solids of capsicum, respectively. At 15th day of storage, result exhibited that the total soluble solids of carrots in T_V0_ show 5.73 ± 0.01 Brix, whereas treatments T_VNP_ and T_VNSP_ expressed 6.16 ± 0.01 and 6.28 ± 0.01 total soluble solids of capsicum respectively. Similarly, Ibukunoluwa, Olawuyi, and Wonyoung (2019) and Gonzalo et al. (2020) evaluated that the total soluble solids of capsicum ranges from 6.66 to 7.50 Brix. During storage (0 and 15 days), T_V0_ acquired a highly decrease in total soluble solids value on 15th day of storage interval, and treatments T_VNP_ and T_VNSP_ showed minor decrease in the total soluble solids of capsicum because nanoparticles and silver nanoparticles preserve the total soluble solids of carrots as fresh during storage. Thus, these two treatments indicate the best storage of capsicum with better shelf life. During this study, with respect to treatments, total soluble solids of capsicum ranged from 6.70 to 6.76 Brix. However, with respect to days, total soluble solids of capsicum was found 6.78 Brix at 0 day and its value decreased at 15th day to 6.70 Brix.

##### 3.2.4.5 Total Soluble Solids of Cucumber

Mean values for total soluble solids of cucumber are exposed in Table 5. Triplicate samples of vegetables were used for each treatment, and total soluble solid was analyzed at 0 day (fresh) and at 15th day of storage. At 0 day, results showed that the total soluble solid value of T_V0_ (control) expressed 2.90 ± 0.02 Brix, whereas treatments T_VNP_ (vegetables with nanoparticles) and T_VNSP_ (vegetables with silver nanoparticles) 2.95 ± 0.01 Brix and 3.00 ± 0.01 Brix total soluble solids of cucumber, respectively. At 15th day of storage, result exhibited that the total soluble solids of cucumber in T_V0_ showed 2.67 ± 0.01 Brix, whereas treatments T_VNP_ and T_VNSP_ expressed 2.90 ± 0.01 Brix and 2.95 ± 0.01 Brix total soluble solids of cucumber, respectively. Similarly, Ibukunoluwa, Olawuyi, and Wonyoung (2019) evaluated that the total soluble solid content of cucumber ranged from 2.45 to 3.30 Brix. During the storage time period (0 and 15 days), T_V0_ acquired a highly decrease in total soluble solid value on 15th day of storage interval, and treatments T_VNP_ and T_VNSP_ showed minor decrease in the total soluble solids of cucumber because nanoparticles and silver nanoparticles preserve the total soluble solids of carrots as fresh during storage, so these two treatments indicate the best storage of cucumber with better shelf life. During this study, with respect to treatments, total soluble solids of cucumber ranged from 3.10 to 3.25 Brix. However, with respect to days, total soluble solids of cucumber is 13.00 Brix at 0 day and the value decreases at 15th day to 11.56 Brix.

#### 3.2.5 Color Analysis of Fruits (Apples and Tomatoes) and Vegetables (Carrots, Capsicum, and Cucumber)

##### 3.2.5.1 Color Values of Apples

Mean values for L*, a*, and b* values of apples are exposed in Table 6. Triplicate samples of fruits were used for each treatment, and L*, a*, and b* value of apples were analyzed at 0 day (fresh) and at 15th day of storage. At 0 day, results showed that L*, a*, and b* values of apples of T_F0_ (control) expressed 48.32% ± 0.02%, 15.12% ± 0.02%, and 11.12% ± 0.02%, respectively, whereas L*, a*, and b* values of treatments T_FNP_ (fruits with nanoparticle) 50.00% ± 0.01%, 18.17% ± 0.01%, and 13.72% ± 0.01%, and T_FSNP_ (fruits with silver nanoparticles) 53.14% ± 0.01%, 21.11% ± 0.01%, and 15.14% ± 0.01%, respectively. At 15th day, results shows that L*, a*, and b* values of apples of T_F0_ (control) expressed 46.67% ± 0.02%, 10.17% ± 0.02%, and 9.67% ± 0.02%, respectively, whereas L*, a*, and b* values of treatments T_FNP_ (fruits with nanoparticles) 48.00% ± 0.01%, 15.00% ± 0.01%, and 11.10% ± 0.01% and T_FSNP_ (fruits with silver nanoparticles) 52.95% ± 0.01%, 18.05% ± 0.01%, and 14.05% ± 0.01%, respectively. However, the results of the present study regarding color values of apples are supported by the previous findings of Dobrzañski and Rybczyñski (2020). During this study, with respect to treatments, color values of L*, a*, and b* expressed increasing to decreasing trends. However, with respect to days, color values of apples L*, a*, and b* was found to be highest at 0 day and their values decreased at 15th day, respectively.

**TABLE 6:** Effect of treatments and days on color (%) of apples.

Treatment	MS						Mean		
	0			15					
	L*	a*	b*	L*	a*	b*	L*	a*	b*
T_F0_	48.32 ± 0.02d	15.12 ± 0.02d	11.12 ± 0.02d	46.67 ± 0.01a	10.17 ± 0.01a	9.67 ± 0.01a	20.00a	130.00a	32.00a
T_FNP_	50.00 ± 0.01e	18.17 ± 0.01e	13.72 ± 0.01e	48.00 ± 0.01b	15.00 ± 0.01b	11.10 ± 0.01b	18.83b	11.83b	30.83b
T_FSNP_	53.14 ± 0.01f	21.11 ± 0.01f	15.14 ± 0.01f	52.05 ± 0.01c	18.05 ± 0.01c	14.05 ± 0.01c	16.00c	10.00c	28.00
Mean	52.56	23.56b	17.56b	51.00a	20.00a	14.00a			

Abbreviations: *T*_V0_ = vegetables control; *T*_VNP_ = vegetables with nanoparticle; *T*_VNSP_ = vegetables with silver nanoparticle.

##### 3.2.5.2 Color Values of Tomatoes

Mean values for L*, a*, and b* values of tomatoes are exposed in Table 7. Triplicate samples of fruits were used for each treatment, and L*, a*, and b* value of tomatoes analyzed at 0 day (fresh) and at 15th day of storage. At 0 day results showed that L*, a*, and b* value of tomatoes of T_F0_ (control) expressed 8.32% ± 0.02%, 4.10% ± 0.02% and 8.70% ± 0.02% respectively. Whereas L*, a*, and b* values of treatments T_FNP_ (fruits with nanoparticle) 10.67% ± 0.01%, 5.30% ± 0.01% and 8.63% ± 0.01% and T_FSNP_ (fruits with silver nanoparticle) 12.14% ± 0.01%, 6.67% ± 0.01% and 8.54% ± 0.01% respectively. At 15th day results shows that L*, a*, and b* value of tomatoes of T_F0_ (control) expressed 6.67% ± 0.02%, 4.00% ± 0.02% and 8.60% ± 0.02% respectively. Whereas L*, a*, and b* values of treatments T_FNP_ (fruits with nanoparticle) 8.50% ± 0.01%, 5.00% ± 0.01% and 8.58% ± 0.01% and T_FSNP_ (fruits with silver nanoparticle) 11.05% ± 0.01%, 6.05% ± 0.01% and 8.50% ± 0.01% respectively. However, the results of present study regarding color value of apples are supported by the previous findings of Dobrzañski and Rybczyñski (2020). During this study, with respect to treatments color values of tomatoes L*, a*, and b* expressed increasing to decreasing trends. However, with respect to days, color values of tomatoes L*, a*, and b* values of tomatoes was found at highest at 0 day and their values decreased at 15th day, respectively.

**TABLE 7:** Effect of treatments and days on color (%) of tomatoes.

Treatment	MS						Mean		
	0			15					
	L*	a*	b*	L*	a*	b*	L*	a*	b*
*T*_F0_	8.32 ± 0.02d	4.10 ± 0.02d	8.70 ± 0.02d	6.67 ± 0.01a	4.00 ± 0.01a	8.60 ± 0.01a	10.00a	8.00a	30.00a
T_FNP_	10.67 ± 0.01e	5.30 ± 0.02d	8.63 ± 0.01e	8.50 ± 0.01b	5.00 ± 0.01b	8.58 ± 0.01b	9.83b	7.83b	28.83b
T_FSNP_	12.14 ± 0.01f	6.67 ± 0.01e	8.54 ± 0.01f	11.05 ± 0.01c	6.05 ± 0.01c	8.50 ± 0.01c	8.00c	5.00c	26.00c
Mean	13.56b	7.20 ± 0.01f	21.50b	10.00a	6.00a	17.00a			

Abbreviations: T_V0_ = vegetables control; T_VNP_ = vegetables with nanoparticle; T_VNSP_ = vegetables with silver nanoparticle.

##### 3.2.5.3 Color Values of Carrots

Mean values for L*, a*, and b* value of carrots are exposed in Table 8. Triplicate samples of fruits were used for each treatment, and L*, a*, and b* values of carrots were analyzed at 0 day (fresh) and at 15th day of storage. At 0 day, results showed that L*, a*, and b* values of carrots of T_V0_ (control) expressed 47.12% ± 0.02%, 13.32% ± 0.01%, and 11.12% ± 0.02%, respectively, whereas L*, a*, and b* values of treatments T_VNP_ (vegetables with nanoparticles) 49.17% ± 0.01%, 15.17% ± 0.01, and 13.43% ± 0.01% and T_VSNP_ (vegetables with silver nanoparticles) 50.00% ± 0.01%, 17.12% ± 0.01% and 16.05% ± 0.01% respectively. The present findings of L* value are in confirmation with the results of Romelle, Rani, and Manohar (2016) stated in the earlier study that L* value of carrots was ranged from 50.00% to 56.10%. At 15th day results shows that L*, a*, and b* value of carrots of T_V0_ (control) expressed 43.00 ± 0.02, 10.17 ± 0.02, and 8.00 ± 0.02, respectively, whereas L*, a*, and b* values of treatments T_VNP_ (vegetables with nanoparticles) 47.00% ± 0.01%, 13.00% ± 0.01%, and 11.00% ± 0.01% and T_VSNP_ (vegetables with silver nanoparticles) 48.05% ± 0.01%, 15.00% ± 0.01%, and 15.02% ± 0.01%, respectively. During this study, with respect to treatments color values of carrots L*, a*, and b* expressed increasing to decreasing trends. However, with respect to days, color values of carrots L*, a*, and b* values of capsicum were found at highest at 0 day and their values decreased at 15th day, respectively.

**TABLE 8:** Effect of treatments and days on color (%) of carrots.

Treatment	MS						Mean		
	0			15					
	L*	a*	b*	L*	a*	b*	L*	a*	b*
T_V0_	47.12 ± 0.02d	13.32 ± 0.02	11.12 ± 0.02d	43.00 ± 0.01a	10.17 ± 0.01a	8.00 ± 0.01a	43.00a	15.00a	40.00a
T_VNP_	49.17 ± 0.01e	15.17 ± 0.01	13.43 ± 0.01e	47.00 ± 0.01	13.00 ± 0.01b	11.00 ± 0.01b	32.83b	13.83b	36.13b
T_VSNP_	50.00 ± 0.01f	17.12 ± 0.01f	16.41 ± 0.01	48.05 ± 0.01c	15.00 ± 0.01c	15.02 ± 0.01c	28.00c	11.00c	34.00c
Mean	22.16b	20.51b	20.32b	20.00a	15.00a	16.00a			

Abbreviations: T_V0_ = vegetables control; T_VNP_ = vegetables with nanoparticle; T_VNSP_ = vegetables with silver nanoparticle.

##### 3.2.5.4 Color Values of Capsicum

Mean values for L*, a*, and b* values of capsicum are shown in Table 9. Triplicate samples of fruits were used for each treatment, and L*, a*, and b* values of capsicum were analyze at 0 day (fresh) and at 15th day of storage. At 0 day, results showed that L*, a*, and b* values of capsicum of T_V0_ (control) expressed 13.00% ± 0.02%, 12.21% ± 0.02%, and 19.12% ± 0.02%, respectively, whereas L*, a*, and b* values of treatments T_VNP_ (vegetables with nanoparticle) 12.05% ± 0.01%, 18.67% ± 0.01%, and 14.00% ± 0.01% and T_VSNP_ (vegetables with silver nanoparticle) 14.00% ± 0.01%, 18.67% ± 0.01%, and 14.00% ± 0.01%, respectively. At 15th day, results show that L*, a*, and b* values of capsicum of T_V0_ (control) expressed 14.00% ± 0.02%, 20.00% ± 0.02%, and 18.00% ± 0.02%, respectively, whereas L*, a*, and b* values of treatments T_VNP_ (vegetables with nanoparticles) 14.00% ± 0.01%, 20.00% ± 0.01%, and 18.00% ± 0.01% and T_VSNP_ (vegetables with silver nanoparticles) 15.05% ± 0.01%, 22.05% ± 0.01%, and 20.05% ± 0.01% respectively. The present findings of color value is in confirmation with the results of Romelle, Rani, and Manohar (2016). During this study, with respect to treatments color values of capsicum L*, a*, and b* expressed increasing to decreasing trends. However, with respect to days, color values of capsicum L*, a*, and b* values of capsicum was found at highest at 0 day and its values decreased at 15th day, respectively.

**TABLE 9:** Effect of treatments and days on color (%) of capsicum.

Treatment	MS						Mean		
	0			15					
	L*	a*	b*	L*	a*	b*	L*	a*	b*
T_V0_	13.00 ± 0.02d	21.12 ± 0.02d	19.12 ± 0.02d	12.05 ± 0.01a	18.67 ± 0.01a	14.00 ± 0.01a	15.00a	20.00a	21.00a
T_VNP_	16.13 ± 0.01	23.17 ± 0.01e	21.67 ± 0.01e	14.00 ± 0.01b	20.00 ± 0.01	18.00 ± 0.01b	13.83b	23.13b	18.13b
T_VSNP_	19.14 ± 0.01f	25.13 ± 0.01f	22.11 ± 0.01f	15.05 ± 0.01c	22.05 ± 0.01c	20.05 ± 0.01c	12.00c	25.00c	16.00c
Mean	13.16b	12.16b	22.00	11.00a	10.00a	18.00a			

Abbreviations: T_V0_ = vegetables control; T_VNP_ = vegetables with nanoparticle; T_VNSP_ = vegetables with silver nanoparticle.

##### 3.2.5.5 Color Values of Cucumber

Mean values for L*, a*, and b* values of cucumber are presented in Table 10. Triplicate samples of fruits were used for each treatment, and L*, a*, and b* values of cucumber were analyzed at 0 day (fresh) and at 15th day of storage. At 0 day, results showed that L*, a*, and b* values of cucumber of T_V0_ (control) expressed 75.30% ± 0.02%, 14.12% ± 0.02%, and 24.00% ± 0.02%, respectively, whereas L*, a*, and b* values of treatments T_VNP_ (vegetables with nanoparticle) 76.67% ± 0.01%, 15.00% ± 0.01%, and 25.17% ± 0.01% and T_VSNP_ (vegetables with silver nanoparticles) 77.56% ± 0.01%, 16.85% ± 0.01%, and 62.25% ± 0.01%, respectively. At 15th day, results show that L*, a*, and b* values of cucumber of T_V0_ (control) expressed 12.17% ± 0.02%, 22.20% ± 0.02%, and 13.00% ± 0.02%, respectively, whereas L*, a*, and b* values of treatments T_VNP_ (vegetables with nanoparticles) 75.00% ± 0.01%, 13.10% ± 0.01%, and 24.00% ± 0.01% and T_VSNP_ (vegetables with silver nanoparticles) 76.05% ± 0.01%, 15.15% ± 0.01%, and 27.10% ± 0.01%, respectively. The current results are interrelated with the previous findings of Zarmeena et al. (2017), During this study, with respect to treatments color values of cucumber L*, a*, and b* expressed increasing to decreasing trends. However, with respect to days, color values of cucumber L*, a*, and b* values of capsicum was found at highest at 0 day and its values decreased at 15th day, respectively.

**TABLE 10:** Effect of treatments and days on color (%) of cucumber.

Treatment	MS						Mean		
	0			15					
	L*	a*	b*	L*	a*	b*	L*	a*	b*
T_V0_	75.30 ± 0.02d	14.12 ± 0.02d	24.00 ± 0.02	73.17 ± 0.01a	12.17 ± 0.01a	22.20 ± 0.01a	13.00a	40.00a	19.00a
T_VNP_	76.67 ± 0.01e	15.00 ± 0.01e	25.17 ± 0.01e	75.00 ± 0.01b	13.10 ± 0.01b	24.00 ± 0.01b	11.83b	37.83	16.13
T_VSNP_	77.56 ± 0.01f	16.85 ± 0.01f	26.25 ± 0.01f	76.05 ± 0.01c	15.15 ± 0.01c	27.10 ± 0.01	10.00c	33.00	14.10c
Mean	20.00b	15.16b	20.11b	18.00a	13.00a	16.00a			

Abbreviations: T_V0_ = vegetables control; T_VNP_ = vegetables with nanoparticle; T_VNSP_ = vegetables with silver nanoparticle.

#### 3.2.6 Visual Decay of Fruits and Vegetables

##### 3.2.6.1 Visual Decay of Fruits (Apples and Tomatoes)

**FIGURE 2:**
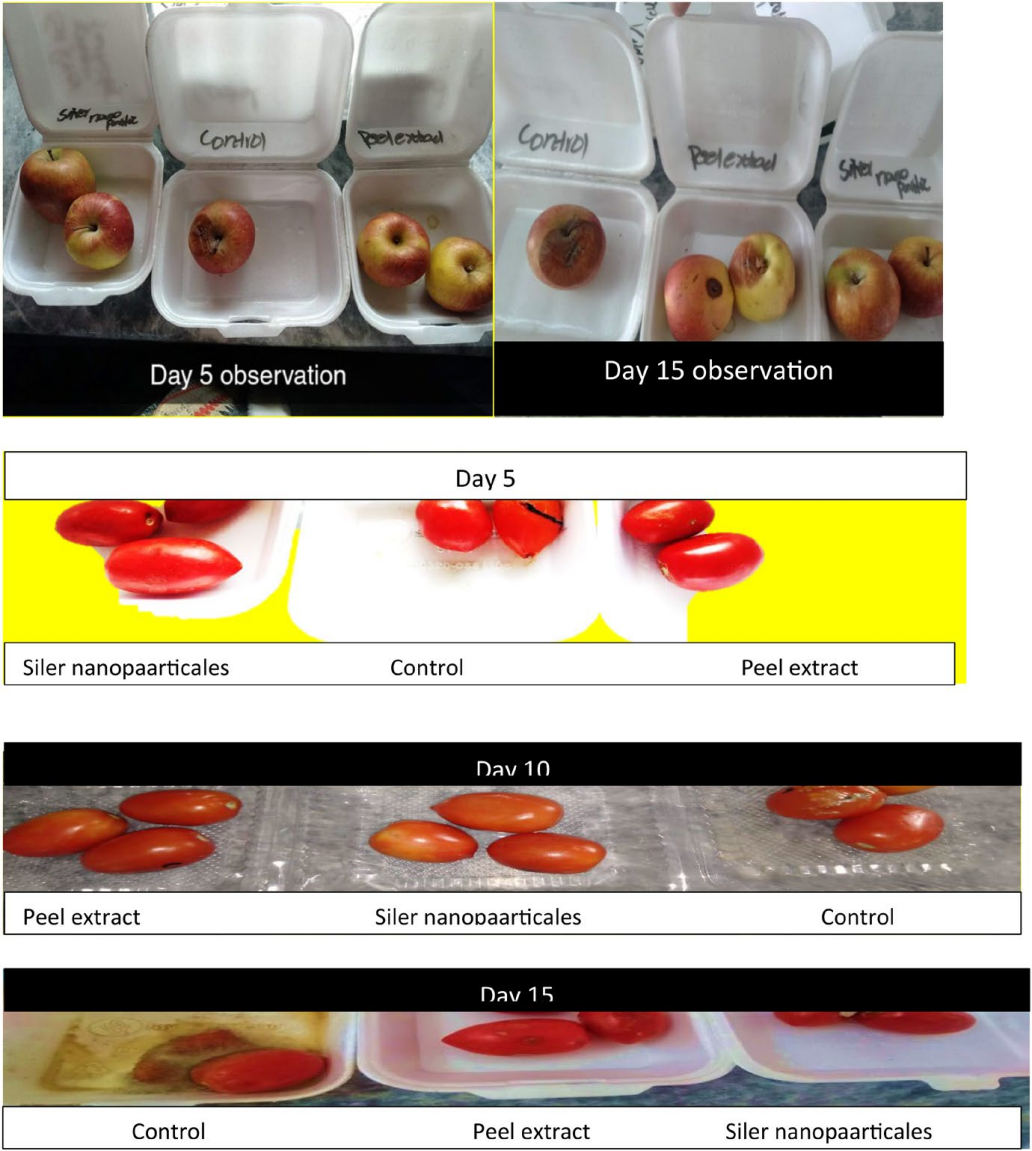
Visual decay of fruits.

Visual decay of apples and tomatoes is presented in Table 11 and Figure 2. Triplicate samples of each fruit were used for each treatment and visual decay of apples and tomatoes was analyzed at 0 day (fresh) and at 15th day of storage. At 0 day observations of visual decay of apples and tomatoes indicated that T_F0_ (control) T_FNP_ (fruits with nanoparticle) and T_FSNP_ (fruits with silver nanoparticle) expressed fresh qualities of fruits. At 15th day observations of visual decay of apples and tomatoes indicated that T_F0_ (control) expressed dull color, wrinkled color, rotting, spotting, fungus, and unacceptable variables occurred, whereas treatments T_FNP_ (fruits with nanoparticles) and T_FSNP_ (fruits with silver nanoparticle) of apples and tomatoes showed almost maintained the quality of fruits because the coating of nanoparticles and silver nanoparticles preserve qualities of the fruits as fresh during storage, so these two treatments indicate the best storage of fruits with better shelf life. The same observations reported by Mohamed, Mahmoud, and Mahmoud (2019) the coating treatments reduce the decay percentage during storage time periods compared to the samples that are uncoated.

**TABLE 11:** Visual decay of fruits and vegetables.

Treatment	Apples and tomatoes												Carrots, capsicum, and cucumber											
	Dull color		Wrinkled		Rotting		Spotting		Fungus		Unacceptable		Dull color		Wrinkled	Rotting			Spotting		Fungus		Unacceptable	
	0	15	0	15	0	15	0	15	0	15	0	15	0	15	0	15	0	15	0	15	0	15	0	15
T_F0_	✗	✓	✗	✓	✗	✓	✗	✓	✗	✓	✗	✓	✗	✓	✗	✓	✗	✓	✗	✓	✗	✓	✗	✓
T_FNP_	✗	✓	✗	✗	✗	✓	✗	✗	✗	✓	✗	✓	✗	✗	✗	✗	✗	✓	✗	✓	✗	✗	✗	✓
T_FSNP_	✗	✗	✗	✗	✗	✗	✗	✗	✗	✗	✗	✗	✗	✓	✗	✗	✗	✗	✗	✗	✗	✗	✗	✗

Abbreviations: T_V0_ = vegetables control; T_VNP_ = vegetables with nanoparticle; T_VNSP_ = vegetables with silver nanoparticle.

##### 3.2.6.2 Visual Decay of Vegetables (Carrots, Capsicum, and Cucumber)

**FIGURE 3:**
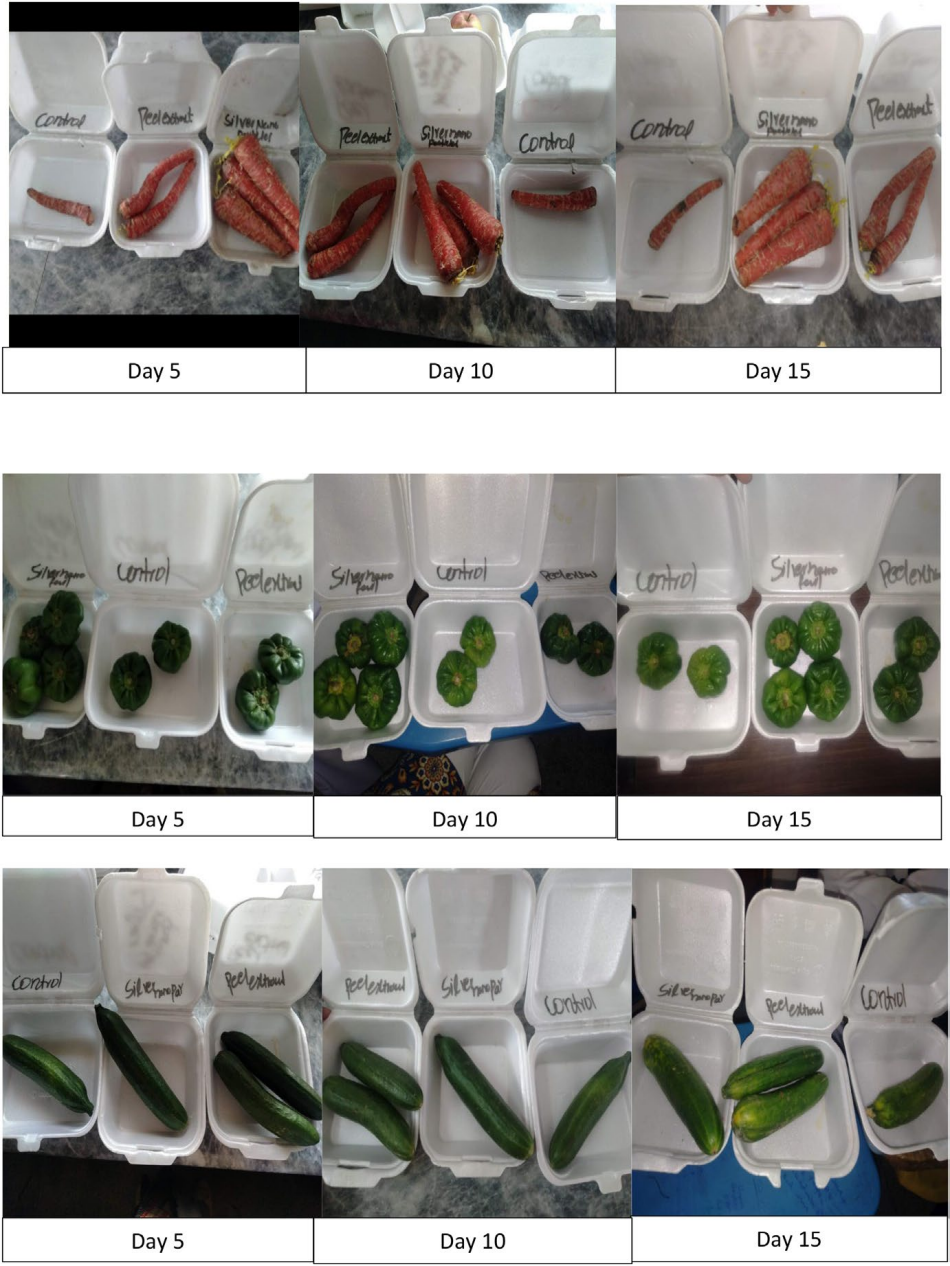
Visual decay of vegetables.

Visual decay of carrots, capsicum, and tomatoes is presented in Table 11 and Figure 3. Triplicate samples of each vegetable were used for each treatment, and visual decay of carrots, capsicum, and cucumber was analyzed at 0 day (fresh) and at 15th day of storage. At 0 day, observations of visual decay of carrots, capsicum, and cucumber indicated that T_V0_ (control) T_VNP_ (vegetables with nanoparticles) and T_VSNP_ (vegetables with silver nanoparticles) expressed fresh qualities of vegetables. At 15th day, observations of carrots, capsicum, and cucumber visual decay indicated that T_V0_ (control) expressed dull color, wrinkled texture, rotting, spotting, fungus, and unacceptable variables occurs, whereas treatments T_FNP_ (vegetables with nanoparticles) and T_VSNP_ (vegetables with silver nanoparticles) of carrots, capsicum, and cucumber showed almost maintained the quality of vegetables because coating of nanoparticles and silver nanoparticles preserve qualities of vegetables as fresh during storage, so these two treatments indicate the best storage of vegetables with better shelf life. The same observations were reported by Sahat et al. (2020) compared to the uncoated sample, the coating treatments decreased the percentage of deterioration during storage.

#### 3.2.7 Weight Loss of Fruits (Apples and Tomatoes) and Vegetables (Carrots, Capsicum and Cucumber)

##### 3.2.7.1 Weight Loss of Apples

Mean values for weight loss of apples are exposed in Table 12. Triplicate samples of fruits were used for each treatment and weighed at 0 day and after every 5th day. Weight loss depends upon transpiration of fruit. At 0 day, results showed that the weight loss in T_F0_ was 16.38 ± 0.04 g, whereas treatments T_FNP_ (fruits with nanoparticle) and T_FSNP_ (fruits with silver nanoparticle) expressed 18.47 ± 0.02 and 20.37 ± 0.02 g weight loss, respectively. At 5th day of storage, results showed that the weight loss in T_F0_ was 13.47 ± 0.04 g, whereas treatments T_FNP_ and T_FSNP_ expressed 16.68 ± 0.02 and 18.40 ± 0.01 g weight loss, respectively. At 10th day of storage, results showed that the weight loss in T_F0_ show 10.78 ± 0.04 g, whereas treatments T_FNP_ and T_FSNP_ expressed 14.75 ± 0.02 and 16.18 ± 0.02 g weight loss, respectively. At 15th day of storage, results showed that the weight loss in T_F0_ show 7.05 ± 0.04 g, whereas treatments T_FNP_ and T_FSNP_ expressed 12.86 ± 0.01 and 14.18 ± 0.04 g weight loss, respectively. During storage from day 0 to day 15, T_F0_ obtained the maximum weight loss values on the 0th, 5th, 10th, and 15th day of storage intervals, whereas T_FSNP_ showed the lowest weight loss value on the 15th day of storage time period; this treatment reveals the best way to store apples. T_FSNP_ had the lowest weight loss score during this trial when it came to treatments, followed by T_FNP_. T_F0_, however, had the greatest weight loss score. In terms of days while being storage, weight loss express decreasing to increasing trend of weight loss in all treatments with the passage of time. During storage at room temperature, the weight loss of treatment T_F0_ (control) show increasing trend compared to other two treatments (T_FNP_ and T_FSNP_) because these two treatments were treated with nanoparticles and silver nanoparticles, respectively, which prevent severe weight loss of apples as storage time proceeds to 15 days. Storage conditions significantly impacted the weight of the fruit. Fruits that have lost weight generally appears shriveled and unappealing (Mishra et al. 2017).

**TABLE 12:** Effect of treatments and days on weight loss (g) score of fruits.

Treatments	Apples					Tomatoes				
	Days				Mean	Days				Mean
	0	5	10	15		0	5	10	15	
T_F0_	16.38 ± 0.04b	13.47 ± 0.04a	10.78 ± 0.04c	7.05 ± 0.04a	21.03c	16.84 ± 0.04a	10.46 ± 0.02c	8.94 ± 0.01a	6.96 ± 0.01a	20.308a
T_FNP_	18.47 ± 0.02c	16.68 ± 0.02b	14.75 ± 0.02c	12.86 ± 0.01b	19.19b	18.81 ± 0.04b	16.74 ± 0.04d	14.97 ± 0.02b	12.97 ± 0.01b	18.02b
T_FSNP_	20.37 ± 0.02a	18.40 ± 0.01c	16.18 ± 0.02f	14.18 ± 0.04c	17.15a	20.84 ± 0.02c	18.10 ± 0.06c	16.18 ± 0.04c	14.18 ± 0.01c	16.38c
Mean	10.07a	14.18c	18.24b	22.74d		12.70a	14.98b	16.10c	18.50d	

Abbreviations: T_V0_ = vegetables control; T_VNP_ = vegetables with nanoparticle; T_VNSP_ = vegetables with silver nanoparticles.

##### 3.2.7.2 Weight Loss of Tomatoes

Mean values for weight loss of tomatoes are exposed in Table 12. Triplicate samples of fruits were used for each treatment and weighed at 0 day and after every 5th day. Weight loss depends upon transpiration of fruit. At 0 day, results showed that the weight loss in T_F0_ was 16.84 ± 0.04 g, whereas treatments T_FNP_ (fruits with nanoparticles) and T_FSNP_ (fruits with silver nanoparticles) expressed 18.81 ± 0.04 and 20.84 ± 0.02 g weight loss, respectively. At 5th days of storage, results showed that the weight loss in T_F0_ was 10.46 ± 0.02 g, whereas treatments T_FNP_ and T_FSNP_ expressed 16.74 ± 0.04 and 18.10 ± 0.06 g weight loss, respectively. At 10th days of storage, results showed that the weight loss in T_F0_ was 8.94 ± 0.01 g, whereas treatments T_FNP_ and T_FSNP_ expressed 14.97 ± 0.02 and 16.18 ± 0.04 g weight loss respectively. At 15th day of storage, results showed that the weight loss in T_F0_ was 6.96 ± 0.01 g, whereas treatments T_FNP_ and T_FSNP_ expressed 12.97 ± 0.01 and 14.18 ± 0.01 g weight loss, respectively. During storage from 0 to 15 days, T_F0_ had shown the highest weight loss values on 0, 5th, 10th, and 15th day's storage intervals, and T_FSNP_ showed lowest weight loss values on the 15th day of storage so this treatment shows the best storage of tomatoes. However, this result was consistent with those reported by Martial‐Didier et al. (2017). With respect to treatments, T_FSNP_ showed the lowest weight loss values followed by T_FNP_. However, T_F0_ showed highest weight loss score. In terms of days while being stored, weight loss expressed a decreasing to increasing trend of weight loss in all treatments with the passage of time. During storage at ambient temperature, the weight loss of treatment T_F0_ (control) showed an increasing trend compared to the other two treatments (T_FNP_ and T_FSNP_) because these two treatments were treated with silver nanoparticles, respectively, which prevent severe weight loss of tomatoes as storage interval proceeds.

##### 3.2.7.3 Weight Loss of Carrots

Mean values for weight loss of carrots are exposed in Table 13. Triplicate samples of vegetables were used for each treatment and weighed at 0 day and after every 5th day. Weight loss depends upon the transpiration rate of vegetables. At 0 day, results showed that the weight loss in T_V0_ was 15.38 ± 0.04 g, whereas treatments T_VNP_ (vegetables with nanoparticles) and T_VNSP_ (vegetables with silver nanoparticles) expressed 18.64 ± 0.01 and 20.19 ± 0.02 g weight loss, respectively. At 5th day of storage, results showed that the weight loss in T_F0_ shows 12.42 ± 0.04 g, whereas treatments T_VNP_ and T_VNSP_ expressed from 16.45 ± 0.02 g to 18.26 ± 0.03 g weight loss, respectively. At 10th day of storage, results showed that the weight loss in T_V0_ shows 8.87 ± 0.04 g, whereas treatments T_VNP_ and T_VNSP_ expressed 14.57 ± 0.02 and 16.65 ± 0.03 g weight loss, respectively. At 15th day of storage, results showed that the weight loss in T_V0_ was 6.88 ± 0.02, whereas treatments T_VNP_ and T_VSNP_ expressed 12.56 ± 0.02 and 14.65 ± 0.04 g weight loss, respectively. During storage from 0 to 15 days, T_V0_ has acquired the highest weight loss values on 0, 5th, 10th, and 15th day, and T_VNSP_ had shown lowest weight loss values on the 15th day of storage, so this treatment shows the best storage of carrots. However, the results of the present study are supported by the previous findings of Varastegani, Zzaman, and Yang (2015). With respect to treatments, T_VNSP_ had shown the lowest weight loss values that were followed by T_VNP_. However, T_V0_ had shown the highest weight loss values. During storage with respect to day's weight loss express a decreasing to increasing trend of weight loss in all treatments with the passage of time. During storage at room temperature, the weight loss of treatment T_V0_ (control) shows an increasing trend compared to the other two treatments (T_VNP_ and T_VNSP_) because these two treatments treated with nanoparticles and silver nanoparticles, respectively, which prevent severe weight loss of carrots as storage time proceeds to 15 days.

**TABLE 13:** Effect of treatments and days on weight loss (g) score of vegetables.

Treatments	Carrots					Capsicum					Cucumber				
	Days				Mean	Days				Mean	Days				Means
	0	5	10	15		0	5	10	15		0	5	10	15	
T_V0_	15.38 ± 0.04i	12.42 ± 0.04g	8.87 ± 0.04c	6.88 ± 0.02a	18.14a	19.84 ± 0.04a	15.95 ± 0.01g	10.57 ± 0.01c	6.61 ± 0.02a	21.99a	15.84 ± 0.04a	13.95 ± 0.01b	11.57 ± 0.02	8.09 ± 0.02a	20.99a
T_VNP_	18.64 ± 0.01	16.45 ± 0.02h	14.57 ± 0.02e	12.56 ± 0.02b	16.06b	21.83 ± 0.01b	18.61 ± 0.04h	14.67 ± 0.02e	12.05 ± 0.01b	19.63b	18.83 ± 0.04b	15.61 ± 0.01c	12.67 ± 0.02b	10.79 ± 0.01b	18.63b
T_VNSP_	20.19 ± 0.02k	18.26 ± 0.03i	16.65 ± 0.03f	14.65 ± 0.04c	13.63c	23.47 ± 0.02c	20.73 ± 0.49i	18.64 ± 0.34	16.06 ± 0.04d	17.56c	20.47 ± 0.02c	18.73 ± 0.03a	16.64 ± 0.01c	14.06 ± 0.04c	15.56c
Mean	10.36a	14.70b	17.38c	19.04d		12.048d	14.436c	18.961b	20.821a		10.04d	15.43c	20.96b	25.82a	

Abbreviations: T_V0_ = vegetables control; T_VNP_ = vegetables with nanoparticle; T_VNSP_ = vegetables with silver nanoparticles.

##### 3.2.7.4 Weight Loss of Capsicum

Mean values for weight loss of capsicum are exposed in Table 13. Triplicate samples of vegetables were used for each treatment and weighed at 0 day and after every 5th day. Weight loss depends on the transpiration rate of vegetables. At 0 day, results showed that the weight loss in T_V0_ was 15.95 ± 0.01 g, whereas treatments T_VNP_ (vegetables with nanoparticles) and T_VNSP_ (vegetables with silver nanoparticles) expressed 21.83 ± 0.01 and 23.47 ± 0.02 g weight loss, respectively. At fifth day of storage, results showed that the weight loss in T_V0_ was 15.95 ± 0.01 g, whereas treatments T_VNP_ and T_VNSP_ expressed 18.61 ± 0.04 and 20.73 ± 0.49 g weight loss, respectively. At 10th day of storage, results showed that the weight loss in T_V0_ was 10.57 ± 0.01 g, whereas treatments T_VNP_ and T_VNSP_ expressed 14.67 ± 0.02 and 18.64 ± 0.34 g weight loss, respectively. At 15th day of storage, result showed that the weight loss in T_V0_ was 6.61 ± 0.02 g, whereas treatments T_VNP_ and T_VNSP_ expressed 12.05 ± 0.01 and 16.06 ± 0.04 g weight loss, respectively. During storage, T_V0_ had the highest weight loss scores, whereas T_VNSP_ had the lowest weight loss scores on the 15th day of storage, so this treatment indicates the best storage of capsicum. Regarding the treatments used in this study, T_VNSP_ likewise had the lowest weight loss score, followed by T_VNP_. T_V0_, however, had the greatest weight loss score. However, this result was consistent with those reported by Martial‐Didier et al. (2017). During storage with respect to day's weight loss express a decreasing to increasing trend in all treatments with the passage of time. During storage at room temperature, the weight loss of treatment T_V0_ (control) shows an increasing trend compared to other two treatments (T_VNP_ and T_VNSP_) because these two treatments treated with nanoparticles and silver nanoparticles, respectively, which prevent severe weight loss of capsicum as storage time proceeds to 15 days.

##### 3.2.7.5 Weight Loss of Cucumber

Mean values for weight loss of cucumber are presented in Table 13. Triplicate samples of vegetables were used for each treatment and weighed at 0 day and after every 5th day. Weight loss depends on the transpiration rate of vegetables. At 0 day, results showed that the weight loss in T_V0_ was 15.84 ± 0.04 g, whereas treatments T_VNP_ (vegetables with nano particles) and T_VNSP_ (vegetables with silver nanoparticles) expressed 18.83 ± 0.04 and 20.47 ± 0.02 g weight loss, respectively. At 5th day of storage, results showed that the weight loss in T_V0_ was 13.95 ± 0.01 g, whereas treatments T_VNP_ and T_VNSP_ expressed 15.61 ± 0.01 and 18.73 ± 0.03 g weight loss, respectively. At 10th day of storage, results showed that the weight loss in T_V0_ was 11.57 ± 0.02 g, whereas treatments T_VNP_ and T_VNSP_ expressed 12.67 ± 0.02 and 16.64 ± 0.01 g weight loss, respectively. At 15th day of storage, the result showed that the weight loss in T_F0_ was 8.09 ± 0.02 g, whereas treatments T_VNP_ and T_VNSP_ expressed 10.79 ± 0.01 and 14.06 ± 0.04 weight loss, respectively. However, the results of the present study are supported by the previous findings of Varastegani, Zzaman, and Yang (2015). During storage (0–15 days), the T_V0_ acquired the highest weight loss scores on day 0, day 5, day 10, and day 15, whereas T_VNP_ showed the lowest value for weight loss score on 15th day of storage, so this treatment shows the best storage of cucumber. With respect to treatments, T_VNSP_ had shown the lowest weight loss value followed by T_VNP_. However, T_V0_ had shown the highest weight loss values. During storage with respect to day's weight loss express a decreasing to increasing trend of weight loss in all treatments with the passage of time. During storage at room temperature, the weight loss of treatment T_V0_ (control) shows an increasing trend compared to the other two treatments (T_VNP_ and T_VNSP_) because these two treatments treated with nanoparticles and silver nanoparticles, respectively, which prevent severe weight loss of cucumber as during storage time for 15 days.

## 4 Conclusions

This study aims to develop silver nanoparticles to mitigate postharvest losses in fruits and vegetables. Research indicates that untreated fruits and vegetables have a shorter shelf life when stored at room temperature, whereas those treated with silver nanoparticles demonstrate a significantly extended shelf life. The use of nanoparticles and silver nanoparticles in apple storage effectively preserves pH, indicating their potential for enhancing the shelf life of fresh produce. This study underscores the benefits of incorporating nanotechnology in food storage practices. The analysis of titratable acidity in apples revealed that treatments with nanoparticles (TFNP) and silver nanoparticles (TFSNP) effectively maintained acidity levels over a 15‐day storage period. Initial acidity values were 1.15 ± 0.03 g/L for the control group (TF0) and increased slightly in treated groups, showing 1.18 ± 0.01 and 1.21 ± 0.01 g/L, respectively. By day 15, while the control group showed a decrease to 1.17 ± 0.02 g/L, both treatments exhibited stability with values of 1.20 ± 0.03 and 1.23 ± 0.01 g/L. This indicates that nanoparticle treatments effectively preserve the titratable acidity of apples, enhancing their shelf life and freshness during storage. Overall, these findings highlight the potential of nanotechnology in improving the quality of stored produce.

The weight loss analysis revealed that the control group (TV0) consistently exhibited the highest weight loss across all storage periods for carrots, capsicum, and cucumber, whereas treatments with nanoparticles (TVNP) and silver nanoparticles (TVNSP) demonstrated significantly lower weight loss, indicating better preservation. Over 15 days, TVNSP showed the least weight loss, suggesting it is the most effective treatment for extending shelf life. These findings highlight the potential of nanoparticle treatments in minimizing weight loss during storage, thereby improving the overall quality and longevity of vegetables. Additionally, combining silver nanoparticles with fruit and vegetable peel extracts may further enhance their longevity. If this approach is successfully introduced to the market following all necessary protocols, it could greatly inspire efforts to reduce postharvest losses and play a crucial role in prolonging the storage life of these products.

## Author Contributions

**Ayesha Shakeel:** conceptualization (equal). **Madiha Rohi:** methodology (equal). **Rizwana Batool:** data curation (equal). **Saima Tehseen:** formal analysis (equal). **Mahwash Aziz:** validation (equal). **Kaynat Malik:** software (equal). **Mahreen Abdul Sattar:** visualization (equal). **Awais Raza:** writing – review and editing (equal). **Agoura Diantom:** investigation (equal), writing – review and editing (equal).

## Disclosure

The authors have nothing to report.

## Consent

All authors agree to publish.

## Conflicts of Interest

The authors declare no conflicts of interest.

## Data Availability

Data supporting the findings of this study are available within the article.
